# Two Novel *De Novo* GARS Mutations Cause Early-Onset Axonal Charcot-Marie-Tooth Disease

**DOI:** 10.1371/journal.pone.0133423

**Published:** 2015-08-05

**Authors:** Yi-Chu Liao, Yo-Tsen Liu, Pei-Chien Tsai, Chia-Ching Chang, Yen-Hua Huang, Bing-Wen Soong, Yi-Chung Lee

**Affiliations:** 1 Department of Neurology, Taipei Veterans General Hospital, Taipei, Taiwan; 2 Department of Neurology, National Yang-Ming University School of Medicine, Taipei, Taiwan; 3 Institute of BioMedical Informatics, National Yang-Ming University School of Medicine, Taipei, Taiwan; 4 Center for Systems and Synthetic Biology, National Yang-Ming University, Taipei, Taiwan; 5 Brain Research Center, National Yang-Ming University, Taipei, Taiwan; Oslo University Hospital, NORWAY

## Abstract

**Background:**

Mutations in the *GARS* gene have been identified in a small number of patients with Charcot-Marie-Tooth disease (CMT) type 2D or distal spinal muscular atrophy type V, for whom disease onset typically occurs during adolescence or young adulthood, initially manifesting as weakness and atrophy of the hand muscles. The role of *GARS* mutations in patients with inherited neuropathies in Taiwan remains elusive.

**Methodology and Principal Findings:**

Mutational analyses of the coding regions of *GARS* were performed using targeted sequencing of 54 patients with molecularly unassigned axonal CMT, who were selected from 340 unrelated CMT patients. Two heterozygous mutations in *GARS*, p.Asp146Tyr and p.Met238Arg, were identified; one in each patient. Both are novel *de novo* mutations. The p.Asp146Tyr mutation is associated with a severe infantile-onset neuropathy and the p.Met238Arg mutation results in childhood-onset disability.

**Conclusion:**

*GARS* mutations are an uncommon cause of CMT in Taiwan. The p.Asp146Tyr and p.Met238Arg mutations are associated with early-onset axonal CMT. These findings broaden the mutational spectrum of *GARS* and also highlight the importance of considering *GARS* mutations as a disease cause in patients with early-onset neuropathies.

## Introduction

Charcot-Marie-Tooth disease (CMT) comprises a clinically, pathologically and genetically heterogeneous group of hereditary neuropathies. Histologically, CMTs are categorized as either demyelinating or axonal according to their electrophysiological or pathological features. Following the advent of molecular genetics, particularly the development of next-generation sequencing, more than 80 disparate mutated genes have been associated with CMT, but only a few of them, including *PMP22*, *GJB1*, *MPZ*, *MFN2* and *NEFL*, account for a significant percentage of CMT cases [1–2]. The rarity with which other mutated genes cause CMT hinders our understanding of their features. Intriguingly, six of the genes responsible for axonal CMT encode aminoacyl- tRNA synthetases (ARSs), which are responsible for charging tRNA molecules with cognate amino acids, suggesting that protein synthesis plays a vital role in axonal functioning. These six genes are glycyl-tRNA synthetase (*GARS*; MIM *600287) [3], tyrosyl-tRNA synthetase (*YARS*; MIM *603623) [4], alanyl-tRNA synthetase (*AARS*; MIM *601065) [5], lysyl-tRNA synthetase (*KARS*; MIM *601421) [6], histidyl-tRNA synthetase (*HARS*; MIM *142810) [7], and methionyl-tRNA synthetase (*MARS*; MIM *156560) [8]. Among them, *GARS* was the first one identified to cause CMT when mutated.

Glycyl-tRNA synthetase protein (GlyRS) is ubiquitously expressed and highly evolutionarily conserved in eukaryotic cells [9]. It is essential for all living cells, including both eukaryotic and prokaryotic cells. The human cytoplasmic GlyRS consists of 685 amino acids, which encode an N-terminal WHEP-TRS domain (Met1- Asp62), a catalytic domain (Asp63-Val556) and a C-terminal anticodon-binding domain (Val557-Glu685) [10], and belongs to the class IIA aminoacyl-tRNA synthetases, which are characterized by three conserved motifs within the catalytic domain [10]. In addition, three insertion domains split the catalytic domain of human GlyRS, including insertion I (Ala145-Asn225), insertion II (His318-Asn349) and insertion III (Val440-Val504) [11]. Human GlyRSs functions as a homodimer, and insertions I and III participate in a series of conformational changes in the human GlyRS homodimer during the aminoacylation of tRNA substrates with glycine [11]. Mutations in *GARS* were firstly reported in 2003, with four missense mutations (p.Glu71Gly, p.Leu129Pro, p.Gly240Arg and p.Gly526Arg) resulting in CMT type 2D (CMT2D; MIM #601472) or distal spinal muscular atrophy type V (dSMA-V; MIM #600794), which is also known as distal hereditary motor neuropathy type VA (dHMN5A) [3]. The clinical presentations of distal hereditary motor neuropathy are similar to CMT except for the absence of sensory involvement. Both CMT2D and dSMA-V (dHMN5A) are usually characterized by disease onset in adolescence or young adulthood and initial manifestations of weakness and atrophy of the hand muscles [3]. In the past 12 years, only 9 more pathogenic mutations in *GARS* have been identified (Table 1) [12–21]. The small number of *GARS* mutations limits our knowledge of the clinical and molecular spectrums of patients with *GARS* mutations. The aim of this study was to ascertain the frequency and spectrum of *GARS* mutations in our cohort of 340 unrelated CMT patients of Han Chinese origin in Taiwan. The clinical presentations of those patients with *GARS* mutations were also characterized.

**Table 1:** Clinical and molecular features of mutations in the *GARS* gene.

Mutation	Protein Structure^a^	Origins	Age of onset^b^	Phenotype^c^	Inheritance^d^	First symptom	References
Ala57Val	W	Ghana	12 y	CMT2D/dSMA-V	Unknown	atrophy and weakness of the hand muscles	[12]
Glu71Gly	C	Monogolia	18 y*	CMT2D/dSMA-V	AD	atrophy and weakness of the hand muscles	[3, 13]
Leu129Pro	C, DI	Bulgaria	16.9 y*	dSMA-V	AD	atrophy and weakness of the hand muscles	[3, 13]
Asp146Asn	Ins I	Korea	15 y	dSMA-V	AD	atrophy and weakness of the hand muscles	[14]
Asp146Tyr	Ins I	Taiwan	<6 m	CMT2	*De novo*	delayed milestones, severe generalized weakness	this study
Ser211Phe	Ins I	Korea	13 y	dSMA-V	AD	atrophy and weakness of the hand muscles	[14]
Leu218Gln	Ins I	Japan	<2 y	CMT2	Unknown	delayed onset of walking, slow running	[15]
Met238Arg	C, DI	Taiwan	2 y	CMT2	*De novo*	delayed onset of walking, unsteady gait	this study
Gly240Arg	C, DI	North America	23 y*	CMT2D	AD	atrophy and weakness of the hand muscles	[3, 13]
Pro244Leu	C	Japan	10 y	CMT2	NA	slow running	[16]
Ile280Phe	C, DI	UK	11–18 y	dSMA-V	AD	distal limb muscle atrophy and weakness	[17]
Ile280Phe	Ins II	USA	34 y	CMT2	AD	progressive unsteadiness walking	[18]
His418Arg	C	UK/Australia	26 y*	dSMA-V	AD	atrophy and weakness of the hand muscles	[13]
Asp500Asn	Ins III	Italy	10–35 y	CMT2D/dSMA-V	AD	atrophy and weakness of the hand muscles	[19]
Gly526Arg	C	Sephardic Jewish	13–26 y	dSMA-V	AD	atrophy and weakness of the hand muscles	[3]
Gly526Arg	C	France	23.3 y*	dSMA-V	AD	atrophy and weakness of the hand muscles or distal four limbs	[20]
Gly598Ala	ACBD	UK	<6 m	Infantile SMA	*De novo*	delayed milestones, severe generalized weakness	[17]
Gly598Ala	ACBD	USA	<6 m	Infantile SMA	*De novo*	delayed milestones, severe generalized weakness	[21]

^a^ C: Catalytic domain; DI: dimer interface; W: WHEP domain; Ins I: Insertion I domain; Ins II: Insertion II domain; Ins III: Insertion III domain; ACBD: anti-codon binding domain

^b^ * average age of disease onset

^c^ CMT: Charcot-Marie-Tooth disease; SMA: spinal muscular atrophy; dSMA: distal SMA;

^d^ AD: autosomal dominant; NA: not available

## Methods

### Patients

Fifty-four patients of Han Chinese descent with molecularly-unassigned CMT2 were recruited in this study. These patients were selected from a consecutive series of 340 unrelated CMT patients at the Neurology Clinics of Taipei Veterans General Hospital who were assessed using standard clinical and electrophysiological evaluations. Among the 340 CMT patients, 103 have axonal CMT (CMT2), with 27 already demonstrated as having a mutation in *GJB1*, *MFN2*, *NEFL*, *HSPB1*, *AARS*, or *TFG*, and another 22 had previously been shown to have no mutation in *GARS* via Sanger sequencing [22,23]. The remaining 54 patients with an age range from 8 to 70 years received *GARS* analysis in this study. CMT2 was diagnosed according to the guidelines described in the report of the 2nd Workshop of the European CMT consortium [24]. Sensory involvement was demonstrated in all the 54 patients by nerve conduction studies (NCS), and the 17p12 duplication or deletion, mutations in the *GJB1* gene or its promoter and mutations in the transthyretin gene had been excluded in advance. Peripheral blood samples were collected after written informed consent was obtained from the participants or their parents on behalf of them for those younger than 18 years. All protocols for this study were approved by the Institutional Review Board of Taipei Veterans General Hospital. The individuals have provided written consent for the use of their information and images as per the consent form for publication in a PLOS Journal.

### Mutation analyses

Genomic DNA was extracted from peripheral blood using standard protocols. A high-throughput targeted sequencing panel covering all coding exons of *GARS* was developed to screen the 54 patients with CMT2. The NimbleGen Design (2.1) online software (http://www.nimblegen.com/products/nimbledesign/) was used to design a library of oligonucleotide probes to capture targeted regions. The NimbleGen SeqCap EZ Choice Library system (Roche NimbleGen, Madison, WI) was used to enrich for custom regions of interest. Following the manufacturer’s instructions, 1 μg of genomic DNA was used for sample library preparation and then hybridized to the capture probes. The final enriched samples were sequenced using the HiSeq2000 platform (Illumina, San Diego, CA). High-throughput sequencing was performed, and the raw image files were processed by Illumina base calling software 1.7 for base calling with default parameters. The sequences of each library were generated as 100-bp paired-end reads. The reference genome for sequence read alignment and variant calling was the Human Genome version 19 (hg19/GRCh37). The BaseSpace pipeline (https://basespace.illumina.com/) was used for variant calling. The Illumina VariantStudio data analysis software (http://variantstudio.software.illumina.com/) was used to annotate variants. All identified *GARS* mutations were validated by Sanger sequencing with intronic primers flanking the coding regions of *GARS* using the Big Dye 3.1 dideoxy terminator method (Applied Biosystems, Foster City, CA) on an ABI Prism 3700 Genetic Analyzer (Applied Biosystems). Amplicon sequences were compared with the reference *GARS* coding sequence (NM_002047.2) and the standard sequence of the short isoform of the human cytoplasmic GlyRS (AAA57001.1) was used to annotate amino-acid changes. Both reference sequences were retrieved from the NCBI Database (http://www.ncbi.nlm.nih.gov). The pathogenic properties of the validated novel variants were further assessed by segregation analysis in patients’ families. Then, the putative pathogenic variants were further discriminated by their absence in 1,000 control chromosomes derived from 500 neurologically healthy individuals of Han Chinese origin recruited at our hospital. The Exome Aggregation Consortium Sequencing Project (ExAC; http://exac.broadinstitute.org) and dbSNP (Build 144; https://www.ncbi.nlm.nih.gov/snp) databases were also queried for these variants. *In silico* prediction of the functional effects of the missense mutations were conducted using Combined Annotation Dependent Depletion (CADD) (http://cadd.gs.washington.edu) [25] and Mutation Taster (http://www.mutationtaster.org) [26]. Evolutionary conservation of the mutation sites was analyzed by aligning amino-acid sequences using the UniProt website (http://www.uniprot.org) [27].

## Results

**Fig 1:**
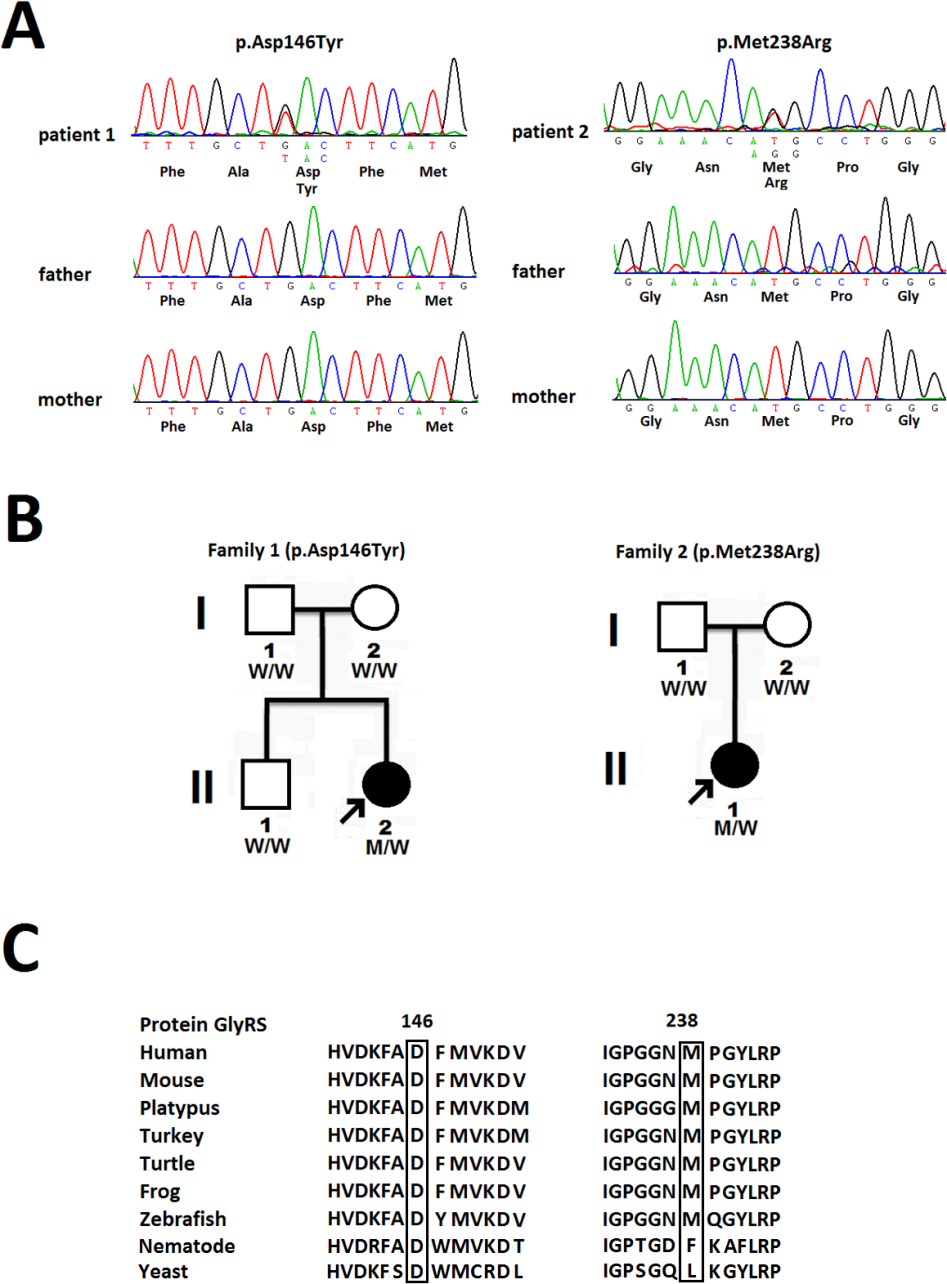
Two novel *de novo GARS* mutations. (A) The novel *GARS* mutations identified in this study, p.Asp146Tyr and p.Met238Arg, with the sense strand electropherogram shown on the top and a limited reading frame depicting the corresponding amino acid substitutions shown below. (B) Pedigrees of the 2 patients harboring the novel *GARS* mutations. The arrows indicate the probands. “M” represents the *GARS* mutations and “W” means wild type. (C) The *GARS* p.Asp146Tyr and p.Met238Arg mutations reside in an evolutionarily conserved region, as shown by aligning the amino acid sequences of glycyl-tRNA synthetase protein (GlyRS) orthologs from various species.

The average coverage and read depth of the target *GARS* sequence are 98% and 516.4X per targeted base, respectively. Mutational analyses of *GARS* in the 54 unrelated patients with CMT2 revealed three heterozygous missense variants, including p.Asp146Tyr (c.598G>T), p.Met238Arg (c.875T>G) (Fig 1A) and p.Asn208Asp (c.784A>G). The last variant was likely a benign polymorphism, as it was absent in two affected siblings of the proband. However, the first two variants, p.Asp146Tyr and p.Met238Arg, are *de novo* mutations (Fig 1B) and are absent in the 61,486 ethnically diverse individuals (122,972 chromosomes) in ExAC, which includes approximately 4,300 individuals from East Asian populations. These mutations are also absent from dbSNP and the 1,000 ethnically matched control chromosomes. Both variants are predicted to be disease-causing mutations by CADD and Mutation Taster programs. The CADD v1.2 PHRED scores for both chr7:30642678G>T and chr7:30649340T>G are 33.0, which place *GARS* p.Asp146Tyr and p.Met238Arg in the top 0.05% most deleterious variants in the genome [25]. Mutation Taster predicted both variants to be disease-causing with strong probability values larger than 0.99; a probability value close to 1 indicates a high “security” of the prediction [26]. The 146th and 238th amino acid residues of the human cytoplasmic GlyRS protein, which is encoded by *GARS*, are also highly evolutionarily conserved (Fig 1C).

**Fig 2:**
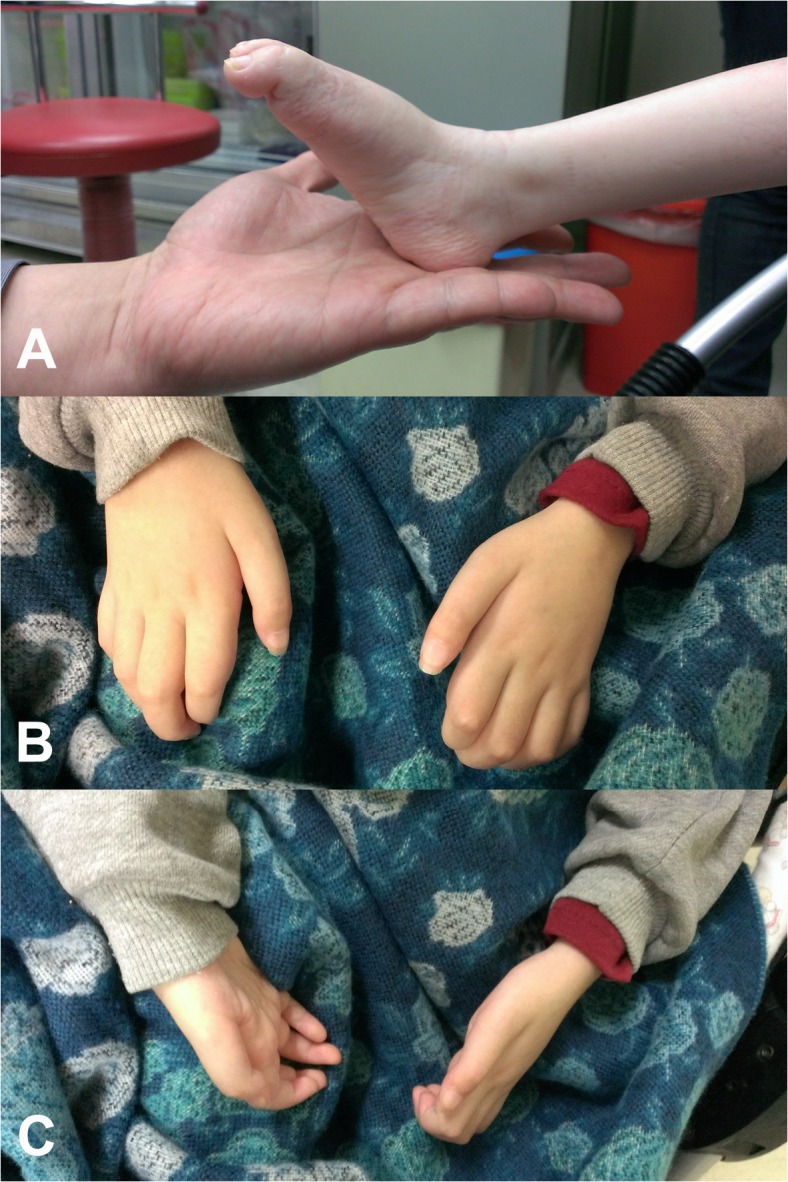
Clinical features of the patient harboring the *GARS* p.Asp146Tyr mutation. (A) Severe weakness and atrophy of the muscles in the feet and legs, pes planus and (B, C) severe intrinsic hand muscle atrophy with claw hand deformity.

Each of the two *de novo GARS* mutations were identified in one single patient, and the paternities between the two index patients and their parents were confirmed by analyzing 14 microsatellite markers. Patient 1 is heterozygous for the *GARS* p.Asp146Tyr mutation. She was reported to have pneumonia with respiratory failure at 3 months of age. Axonal polyneuropathy was diagnosed at that time, and pneumonia with respiratory failure was cured with treatment. The patient had delayed motor milestones and was not able to sit by herself until 15 months of age. She could never stand, pinch, or move her toes. She learned to control an electrical wheelchair by using a joystick at age 7. Neurological examination at age 9 revealed pes planus, generalized areflexia and severe and symmetrical weakness and atrophy of the muscles in four limbs (Fig 2). The Medical Research Council (MRC) scale scores were grade 4 for muscles of the shoulder and pelvic girdles, grade 3 for flexors and extensors of the elbows and knees, grade 1–2 for the wrist muscles and finger flexors, and grade 0 for the intrinsic hand and feet muscles and muscles in the legs. All modalities of sensation were diminished in the regions distal to the knees and wrists. The NCS demonstrated a severe polyneuropathy with a failure to elicit any motor or sensory response in the limbs. Patient 2 is heterozygous for the *GARS* p.Met238Arg mutation. She had no delayed onset of ambulation but began to experience gait difficulty with frequent tripping at age 2. She developed feet drop and difficulty pinching at age 8 and became almost wheelchair-bound at age 11. Neurological examination at age 11 revealed pes cavus, generalized areflexia, and severe weakness and atrophy of the intrinsic feet and hand muscles and the muscles in the legs. The MRC scores were grade 4 for flexors and extensors of the knees, grade 3 for the wrist flexor and extensor and finger flexor muscles, and grade 0 for the intrinsic hand and feet muscles and muscles in the legs. The strength of muscles of the pelvic and shoulder girdles and the elbow flexors or extensors was normal. All modalities of sensation were diminished in the regions distal to the ankles. The NCS demonstrated a severe axonal polyneuropathy with right ulnar nerve motor conduction velocity of 43.5 m/s and a compound motor action potential of 0.1 mV. Other motor or sensory responses in the NCS were absent.

## Discussion

In this study, we screened a Taiwanese cohort of 54 unrelated patients with molecularly unassigned CMT2 for mutations in the *GARS* gene and identified two novel *de novo* missense mutations, p.Asp146Tyr and p.Met238Arg, in two patients with early onset CMT2. In addition to the nature of *de novo* mutation, the pathogenicity of both mutations is supported by the following facts. First, the mutations are absent in the 1,000 ethnically matched control chromosomes and two large genetic polymorphisms databases (i.e., ExAC and dbSNP). Second, the mutations are predicted to be pathogenic by two bioinformatics tools, CADD and Mutation Taster. Third, the p.Asp146Tyr and p.Met238Arg mutations lead to non-conservative substitutions of a negatively charged aspartic acid with a nonpolar tyrosine and a nonpolar methionine with a positively charged arginine at evolutionarily conserved sites in the human GlyRS protein.

**Fig 3:**
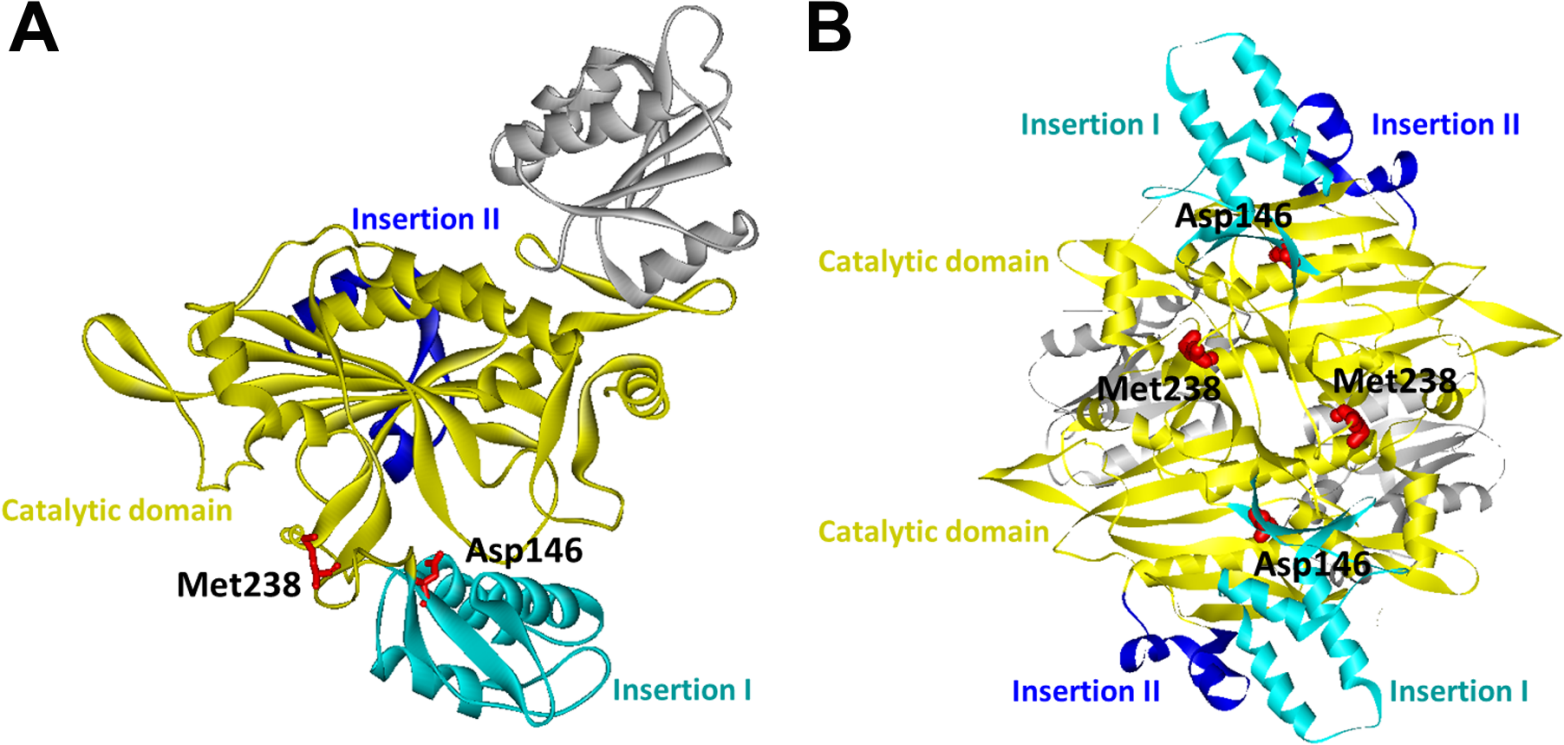
The crystal structure of human glycyl-tRNA synthetase (GlyRS) (PDB ID: 2PME). (A) Mapping of the two *GARS* mutation sites (p.Asp146Tyr and p.Met238Arg) on one subunit of the human GlyRS dimer. (B) A view of the human GlyRS dimers demonstrating that Met238 is located on the interface between the two subunits. Catalytic, Insertion I and Insertion II domains are colored as indicated.

The pathogenic effects of these two mutations appear to be related to their roles in the protein conformational dynamics and homodimeric protein-protein interactions of human GlyRSs. On the basis of the three-dimensional structure of the human GlyRS dimer, Asp146 is located within the insertion I domain and Met238 is situated in the catalytic domain and also at the dimer interface (Fig 3) [10]. Insertion I domain is involved in multiple conformational changes in GlyRS during catalysis, and an altered GlyRS dimer interface has been recognized as a common feature of previously identified CMT-associated GlyRS mutants [10, 11, 28]. Therefore, *GARS* p.Asp146Tyr and p.Met238Arg mutations might cause CMT by compromising the enzymatic function of human GlyRS and influencing its functional conformations or disrupting the dimer interface.

The majority of previously identified *GARS* mutations are linked to CMT2D or dSMA-V and are characterized by autosomal dominant inheritance, disease onset in adolescence or young adulthood and initial clinical manifestation of hand muscle weakness and atrophy (Table 1). Only three of the mutations are associated with early-onset disease. Among them, the *GARS* p.Leu218Gln mutation was found in a patient with CMT2 who had a slightly delayed onset of walking and who developed feet drop at age 7 [15]; p.Gly598Ala mutations were identified in the patients with severe infantile-onset generalized limb muscle weakness and atrophy and a lifetime inability to walk [17,21]. The two novel *GARS* mutations reported here, p.Asp146Tyr and p.Met238Arg, also cause early-onset CMT2 with presentations of severe infantile-onset generalized weakness and early childhood-onset disability, respectively. Our findings stress the importance of considering mutations in *GARS* as a disease cause for patients with early-onset neuropathy.

Interestingly, the *GARS* p.Asp146Tyr mutation occurs at the same residue as the Asp146Asn mutation, which was reported in a Korean dHMN-V family. Korean patients with the Asp164Asn mutation display typical adolescent-onset dHMN-V, whereas our patient with the Asp164Tyr mutation had severe infantile-onset CMT2. The discrepancy between the clinical severities associated with the two mutations at the same residue may result from differences in the biochemical natures of asparagine and tyrosine. Given the rarity of *GARS* mutations, the Asp146 may represent a mutational hot-spot in the *GARS* gene.

The frequency of *GARS* mutations in Taiwanese patients with CMT is low (0.59%; 2/340). In previous studies, Saporta et al. identified three patients with *GARS* mutations from among 787 American patients with CMT (0.4%; 3/787) [29]. Fridman et al. analyzed clinical and genetic data from patients in the Inherited Neuropathies Consortium and found that two out of 1652 CMT patients had *GARS* mutations (0.12%; 2/1652) [30]. After pooling these data, the frequency of *GARS* mutations in CMT patients is approximately 0.25% (7/2779), indicating that *GARS* mutations are an uncommon cause of CMT. This study provides a rare chance to expand our knowledge of the genetic and clinical spectrum of *GARS*–associated neuropathy.

In conclusion, *GARS* p.Asp146Tyr and Met238Arg are novel causes of early- onset axonal CMT. Mutations in *GARS* are uncommon as the cause of CMT in Taiwan. This study illustrates the clinical and genetic features of *GARS* mutations in Taiwan, expands the spectrum of *GARS* mutations and highlights the role of *GARS* mutations in early-onset neuropathy.
